# Effectiveness of insecticidal nets on uncomplicated clinical malaria: a case–control study for operational evaluation

**DOI:** 10.1186/s12936-016-1156-2

**Published:** 2016-02-19

**Authors:** Georgia Barikissou Damien, Armel Djènontin, Evelyne Chaffa, Sandra Yamadjako, Papa Makhtar Drame, Emmanuel Elanga Ndille, Marie-Claire Henry, Vincent Corbel, Franck Remoué, Christophe Rogier

**Affiliations:** UMR MIVEGEC (IRD224-CNRS5290-Universités Montpellier 1 et 2), Institut de Recherche pour le Développement (IRD), 34394 Montpellier, France; IRD-UMR MIVEGEC (IRD224-CNRS5290-Universités Montpellier 1 et 2), Centre de Recherche Entomologique de Cotonou (CREC), Cotonou, Benin; Laboratoire Evolution, Biodiversité des Arthropodes et Assainissement, Faculté des Sciences et Techniques, Université d’Abomey-Calavi, Abomey-Calavi, Bénin; Programme National de Lutte contre le Paludisme (PNLP), Direction Nationale de la Santé Publique, Ministère de la Santé, Cotonou, Benin; Laboratory of Parasitic Diseases, NIAID, NIH, 4 Center Dr, Bethesda, MD 20892-0425 USA; Institut Pasteur de Madagascar, Antananarivo, Madagascar; Institut de Recherche Biomédicale des Armées, Brétigny sur Orge, France; Unité de Recherche sur les Maladies Infectieuses et Tropicales Emergentes, UM 63, CNRS 7278, IRD 198, INSERM 1095, Aix-Marseille Université, Marseille, France

**Keywords:** Effectiveness, Case–control study, Evaluation, Malaria, LLIN

## Abstract

**Background:**

In a context of large-scale implementation of malaria vector control tools, such as the distribution of long-lasting insecticide nets (LLIN), it is necessary to regularly assess whether strategies are progressing as expected and then evaluate their effectiveness. The present study used the case–control approach to evaluate the effectiveness of LLIN 42 months after national wide distribution. This study design offers an alternative to cohort study and randomized control trial as it permits to avoid many ethical issues inherent to them.

**Methods:**

From April to August 2011, a case–control study was conducted in two health districts in Benin; Ouidah–Kpomasse–Tori (OKT) in the south and Djougou–Copargo–Ouake (DCO) in the north. Children aged 0–60 months randomly selected from community were included. Cases were children with a high axillary temperature (≥37.5 °C) or a reported history of fever during the last 48 h with a positive rapid diagnostic test (RDT). Controls were children with neither fever nor signs suggesting malaria with a negative RDT. The necessary sample size was at least 396 cases and 1188 controls from each site. The main exposure variable was “sleeping every night under an LLIN for the 2 weeks before the survey” (SL). The protective effectiveness (PE) of LLIN was calculated as PE = 1 − odds ratio.

**Results:**

The declared SL range was low, with 17.0 and 27.5 % in cases and controls in the OKT area, and 44.9 and 56.5 % in cases and controls, in the DCO area, respectively. The declared SL conferred 40.5 % (95 % CI 22.2–54.5 %) and 55.5 % (95 % CI 28.2–72.4 %) protection against uncomplicated malaria in the OKT and the DCO areas, respectively. Significant differences in PE were observed according to the mother’s education level.

**Conclusion:**

In the context of a mass distribution of LLIN, their use still conferred protection in up to 55 % against the occurrence of clinical malaria cases in children. Social factors, the poor use and the poor condition of an LLIN can be in disfavour with its effectiveness. In areas, where LLIN coverage is assumed to be universal or targeted at high-risk populations, case–control studies should be regularly conducted to monitor the effectiveness of LLIN. The findings will help National Malaria Control Programme and their partners to improve the quality of malaria control according to the particularity of each area or region as far as possible.

## Background

Malaria remains a public health problem in sub-Saharan Africa despite massive control measures deployed over the last several years. The most vulnerable populations are children under 5 years of age [1]. Many countries have focused their efforts on the Roll Back Malaria strategies, such as coverage and use of long-lasting insecticide-treated nets (LLIN), indoor residual spraying (IRS) if appropriate, early case detection with the rapid diagnostic test (RDT), and prompt appropriate treatment by artemisinin-based combination therapy (ACT) [2]. Between 2000 and 2010, the World Health Organization (WHO) recorded reductions of more than 50 % in the health centre-reported cases in 43 of the 99 countries endemic for malaria, showing major progress [3].

LLIN remains the most common and effective tool used to prevent malaria [4]. Between 2004 and 2013, the ownership rate of LLIN strongly increased from 5 to 67 % [4]. Some randomized control studies have already shown that LLIN implementation leads to significant protection against malaria infection, morbidity, and mortality [5, 6]. For the purpose of reducing malaria case incidence rates by 75 % by 2015, operational research is needed to understand effectiveness of vector control interventions [4].

In Benin, large-scale coverage of LLIN using free distribution has been implemented since 2007. Nevertheless the number of reported cases of malaria in 2012 remained high with confirmed 1,365,389 cases in the general population of which 601,347 were children under 5 years of age [7]. The proof of malaria morbidity reduction has become the major objective of the National Malaria Control Programme (NMCP) in Benin, and few studies at the community level have been conducted on this subject [7]. Therefore, some questions still need answers in Benin. For example are malaria control strategies effective? What about LLIN effectiveness in the context of vectors resistance to pyrethroid insecticide? Do the investigators currently have adequate and operational indicators to evaluate malaria control tools such as LLIN in the context of universal coverage?

To evaluate the effectiveness of LLIN, case–control studies offers an alternative to cohort study and randomized control trial as it permits to avoid many ethical issues inherent to them. This type of epidemiological study has already been used for the evaluation of interventions (vaccines, vector control tools) against other infectious diseases, because it avoids many of the ethical issues inherent in longitudinal and experimental studies [8–13]. In the current context of wide distribution of LLIN, it is not possible to compare a group without LLIN coverage and a group with LLIN using a phase III trial. A case–control study can also be less expensive and more operationally feasible than longitudinal studies. In the present study, the main objective was to use a case–control design to evaluate the effectiveness of LLIN on uncomplicated malaria in children, using the main exposure variable defined as “sleeping every night under an LLIN for the 2 weeks before the survey” (SL) and, taking into account epidemiological and social information related to malaria control.

## Methods

### Study design

**Fig. 1:**
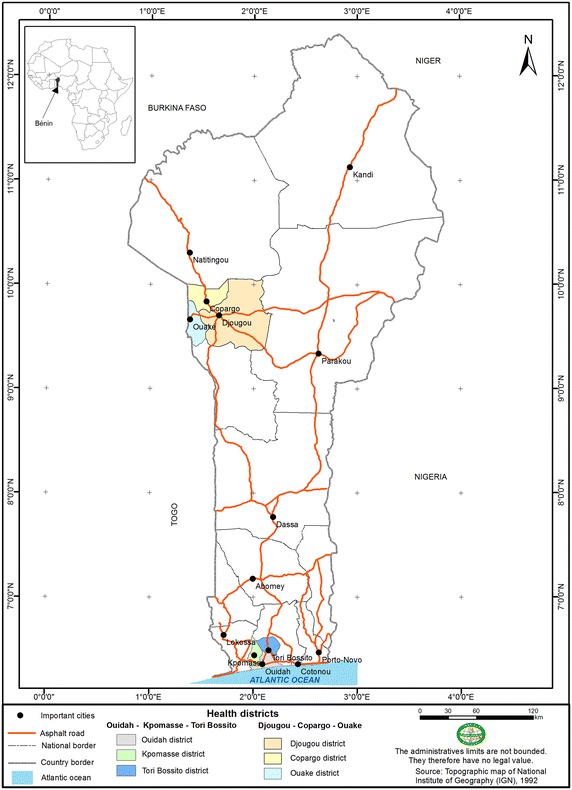
Map of Republic of Benin (RB) located in West Africa. Ouidah–Kpomasse–Tori Bossito and Djougou–Copargo–Ouake health districts were the study area

**Fig. 2:**
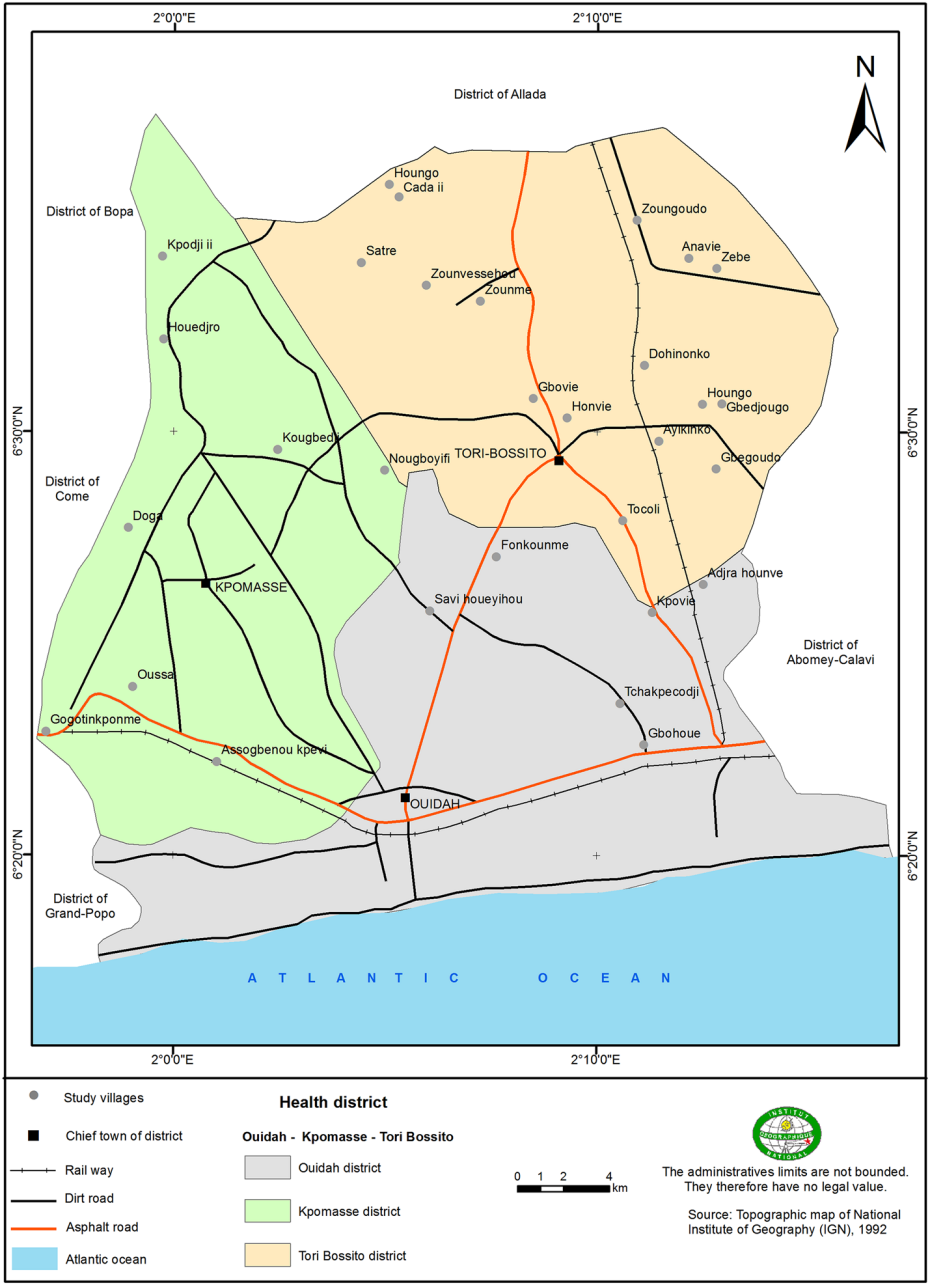
Study villages located in Ouidah–Kpomasse–Tori Bossito health district in southern Benin. A total of 31 villages were included

**Fig. 3:**
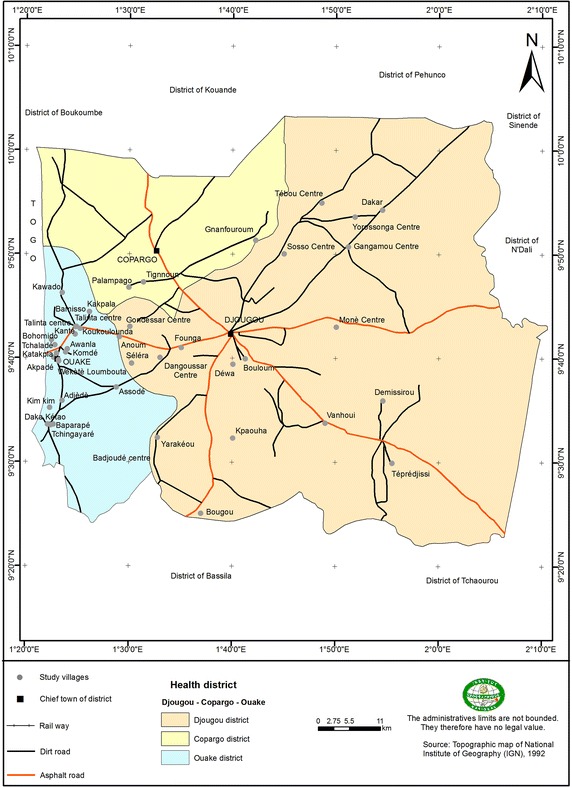
Study villages located in Djougou–Copargo–Ouake health district in northern Benin. A total of 42 villages were included

**Fig. 4:**
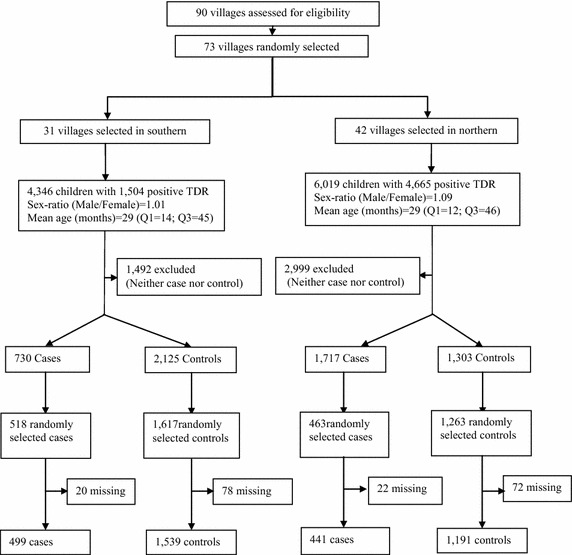
Study profile. *Cases* were children with a high axillary temperature (≥37.5 °C) or a reported history of fever during the last 48 h with a positive rapid diagnostic test (RDT). *Controls* were children with neither fever nor signs suggesting malaria with a negative RDT. *Neither case nor control* were (i) children with a high axillary temperature (≥37.5 °C) or a reported history of fever during the last 48 h with a negative RDT and (ii) children with no signs suggesting malaria associate to positive RDT

A matched case–control study was carried out in the Ouidah–Kpomasse–Tori Bossito (OKT), and Djougou–Copargo–Ouake (DCO) rural health districts located in Benin, West Africa (Fig. 1). In both districts, LLINs were freely distributed to pregnant women and children aged from 0 to 5 years by the NMCP in October 2007 by national campaign. Permanent free distribution has been provided by maternity during pre natal consultations and during vaccination for children aged 9 months. The study area was free of indoor residual spraying campaign. Pyrethroid resistance was widely reported in African malaria vectors especially in Benin [14, 15]. In the study areas (Figs. 2, 3) vector resistance to pyrethroid insecticides was detected simultaneously; the mortality rate of vectors after exposure to permethrin and deltamethrin were 20 and 60 % respectively in OKT, 10 and 36 % in DCO (unpublished data) [16]. The characteristics of these areas have been described previously [16–18]. The inclusion criteria for villages were population size 1000–1800 inhabitants, with the objective of observing at least 15 malaria cases per village. The target population was children aged 0–60 months, living in the selected villages, whose parents gave their informed consent. At least 30 villages were randomly selected in each health district. At first all the children aged 0–60 months were included in the study. After determining the clinical status, “neither case nor control” children were excluded (Fig. 4). Cases and controls were matched by village of residence (1:3). A case was defined as a child with a (i) high axillary temperature (≥37.5 °C) or (ii) a history of fever as reported by the mother or guardian during the 48 h preceding the day of blood sampling, whatever the association with other signs suggesting malaria (sweats, shivers, headaches, nausea, or vomiting) but associated with a positive RDT. A control was defined as a child who had no fever or signs suggesting malaria with a negative RDT. The CareStart™ RDT used detected histidine-rich protein-2 (HPR2) specific to *Plasmodium falciparum*.

### Ethical clearance

Ethics clearance for the study was obtained from the National Ethical Committee for Medical Research in Benin (CNERS, Number 003, 24 March 2011, Institutional Review Board No. 00006860) and Institut de Recherche pour le Développement (IRD)’s consultative committee for deontology and ethics (CCDE). All sick children, whether or not they were participating in the study, were treated by medical staff based on an integrated management of childhood illness strategy during the investigation. Specifically, the malaria cases were treated with artemether–lumefantrine, according to NMCP recommendations in Benin.

### Sample size

The sample size was calculated under the assumption of 30 % protection conferred by using LN, as previously observed in southern Benin in prevalence of infection [18]. The sample size formula used was: N = [p × q (1 + 1/c)(Z_α_ + Z_2β_)^2^] × cluster effect/(p_0_ − p_1_)^2^ [12], where N is the number of cases, p_0_ = 50 % is the proportion of persons using LLIN among the controls, p_1_ = 33 % is the proportion of persons using LLIN among the cases, p = 45.75 % is the proportion of persons using LLIN in the whole sample, q = 1 − p, c = 3 is the number of controls per case, Z_α_ = 1.645 for a unilateral alpha risk of 5 % and Z_2β_ = 0.84 for a power at 80 %, and with a cluster effect of 1.5. Under these assumptions, the required sample size was 396 cases and 1188 controls per site.

### Procedure

Data were collected over 6 weeks in the rainy season (peak of malaria transmission), from April to May 2011 in the OKT district and from July to August 2011 in the DCO district using a cross-sectional survey. Cases and controls were identified by medical staff and by active detection in community in order to identify all children in each village (Figs. 2, 3). CareStart™ RDTs belonged to those certified and recommended by WHO and were assumed to have a 100 % sensitivity and a 100 % specificity for a parasite density at 2000 parasites/µL [19]. All children were tested by RDT. When the result of the RDT was invalid, it was cancelled and re-tested. Cases and controls were randomly selected with equal probability of inclusion per village. The following day after clinical status determination and RDT testing, the mothers or the guardians of the children were questioned face-to-face by trained interviewers to obtain demographic information and determine the use of bed nets and other prevention tools. A standardized questionnaire was administrated under the same conditions for cases and controls by the same investigators to collect information concerning the use of LLIN. Interviewers were not informed of the clinical status of the children. Questionnaires were verbally administrated in French or in the local language. When the mother or a guardian was absent, the team came back the next day to administer the questionnaire. When the mother or guardian was not found the second day, the child was considered as lost to follow-up. Data collection was supervised daily and systematically. An unexpected quality control was periodically undertaken on the data collection in the field and on 10 % of the RDTs.

### Measurement of exposure variables

The principal exposure variable was SL considering the 14-day incubation period of *P. falciparum*. A LLIN was sought and verified *de visu* (in the hung position) by the interviewers during the survey. Two categories of responses were defined, i.e. “always” and “not always” used. The modality “not always” included the answers “sometimes” and “never” used. The analysis also included variables for controlling the following potential confounding factors: use of other tools than LLIN for protection against mosquito bites (coil, domestic insecticide spray, smoking with traditional grass), use of drugs perceived to be malaria chemoprophylaxis during the previous 2 weeks (herbal tea, paracetamol, and chloroquine), child’s age in two groups (0–23 or 24–60 months), sex, the time the child went to bed the previous night (at night fall, before 10 p.m. and after 10 p.m.), mother’s or guardian’s age grouped as <25, 25–35, and >35 years old, mother’s education level (at least primary school or no education), mother’s economic activity, number of dependent children (1, 2, 3, or ≥4), mother’s or guardian’s knowledge of malaria transmission (i.e. malaria is transmitted by mosquito bites) and prevention (i.e. mosquito nets can prevent malaria). The LN condition was assessed by the age of the net from the beginning of its use (≤3 or >3 years), physical condition (with or without holes), and cumulative number of washings (≤20 or >20 washes). The age of the LN was estimated using time references (periods of mass distribution and age of children for whom the LN was given).

### Statistical analysis

All data were entered in the field and checked for errors using the Access 2003 program. The data were analysed using SAS version 9.3 (SAS Institute Inc., Cary, NC, USA). The proportions of cases and controls according to exposure variables were calculated. A sample of 1:3 case–controls matched in the village of residence in the OKT and DCO health districts was considered. The adjusted matched odds ratio (OR) and 95 % confidence interval (CI) for calculation of LN effectiveness were estimated using conditional logistic regression and taking into account the cluster random effect [20, 21]. Variables associated with clinical uncomplicated malaria with a *p* value <0.25 in univariate analysis were included in multivariate analysis. For all of the analysis, the level of significance was set at 0.05. LN effectiveness as protective efficacy (PE) was calculated as: (1 − matched OR) × 100.

## Results

A total of 90 villages were eligible for the study. Seventy-three of them were randomly selected, 31 in the OKT health district and 42 in the DCO district. Details of the study profile are described in Fig. 4. Finally, 499 cases and 1539 controls in the OKT district and 441 cases and 1191 controls in the DCO district were enrolled in the investigation.

The characteristics of the cases and controls included are shown in Tables 1 and 2, according to child, LLIN, and mother or guardian characteristics. Overall, the controls were younger in age than the cases in both health districts (p < 0.0001) with no significant difference according to sex. In the OKT district, 17.0 % of the cases and 27.5 % of the controls had slept under a LLIN every night during the previous 2 weeks compared to 44.9 % of cases and 56.5 % of the controls in the DCO district.

**Table 1:** Factors associated with uncomplicated clinical malaria, Ouidah–Kpomasse–Tori Bossito health district

Variables	N case	N control	Crude OR (95 % CI)	p value
*Information about children*				
Sleeping every night under an LLIN for the 2 weeks before the survey				
Sometimes or never	414	1116	1	
Every night	85	423	0.54 (0.42–0.70)	<0.0001
Age group (month)				
24–60	394	809	1	
0–23	105	730	0.29 (0.23–0.37)	<0.0001
Sex				
Male	252	776	1	
Female	247	763	0.99 (0.82–1.23)	0.9756
Use of drugs perceive to be prophylactic treatment against malaria				
No	126	488	1	
Yes	373	1051	1.37 (1.09–1.73)	0.0063
Hour of sleeping last night				<0.0001
At night fall	354	933	1	
Between night fall and 10 p.m.	123	478	0.68 (0.54–0.86)	0.0011
After 10 p.m.	22	128	0.45 (0.28–0.72)	0.0009
Use other anti-mosquito measures				
Never	317	1109	1	
Every day or sometimes	182	430	1.48 (1.20–1.83)	0.0003
*Information about mosquito net*				
Age (year)				
>3	156	373	1	
≤3	343	1166	0.70 (0.56–0.88)	0.0018
Number of times washed since obtained				
>20	111	333	1	
≤20	388	1206	0.96 (0.76–1.23)	0.7742
Holes				
No	251	744	1	
Yes	248	795	0.92 (0.76–1.13)	0.4444
*Information about mother or guardian*				
Age (year)				0.0004
<25	110	365	1.07 (0.83–1.38)	0.6121
25–35	234	830	1	
>35	155	344	1.60 (1.26–2.03)	0.0001
Primary school at least				
No	365	1048	1	
Yes	134	491	0.78 (0.63–0.98)	0.0337
Profit-making activity				0.4144
Farming/breeding/fishing/handicraft	219	645	1	
Employee status/shopkeeper	269	844	1.54 (0.79–3.01)	0.2046
No activity	11	50	1.45 (0.74–2.82)	0.2761
Number of children in the household				0.0054
1	57	252	1	
2	107	362	1.31 (0.91–1.87)	0.1456
3	105	337	1.38 (0.96–1.98)	0.0834
≥4	230	588	1.73 (1.25–2.40)	0.0010
Knows mosquitoes transmit malaria				
No	10	25	1	
Yes	489	1514	0.81 (0.38–1.70)	0.5727
Knows mosquito net protection against malaria				
No	37	105	1	
Yes	462	1434	0.91 (0.62–1.35)	0.6523

Factors associated with uncomplicated clinical malaria were grouped into information (in italic text) about (i) children aged 0–60 months, (ii) mosquito net, and (iii) mother or guardian of children characteristics. Univariate conditional logistic regression taking into account cluster effect was used

**Table 2:** Factors associated with uncomplicated clinical malaria, Djougou–Copargo–Ouake health district

Variables	N case	N control	Crude OR (95 % CI)	p value
*Information about children*				
Sleeping every night under an LLIN for the 2 weeks before the survey				
Sometimes or never	243	518	1	
Every night	198	673	0.63 (0.50–0.78)	0.0001
Age group (month)				
24–60	293	366	1	
0–23	148	825	0.22 (0.18–0.28)	<0.0001
Sex				
Male	226	637	1	
Female	215	554	1.09 (0.88–1.36)	0.4227
Use of drugs perceive to be prophylactic treatment against malaria				
No	300	871	1	
Yes	141	320	1.28 (1.01–1.62)	0.0418
Hour of sleeping last night				0.2891
At night fall	135	393	1	
Between night fall and 10 p.m.	243	660	1.07 (0.84–1.37)	0.1169
After 10 p.m.	63	138	1.33 (0.93–1.90)	0.5796
Use other anti-mosquito measures				
Never	326	890	1	
Every day or sometimes	115	301	1.04 (0.81–1.34)	0.7401
*Information about mosquito net*				
Age (year)				
>3	86	178	1	
≤3	355	1013	0.73 (0.55–0.96)	0.0269
Number of times washed since obtained				
>20	69	200	1	
≤20	372	991	1.09 (0.81–1.47)	0.5804
Holes				
No	143	360	1	
Yes	298	831	0.90 (0.72–1.14)	0.3893
*Information about mother or guardian*				
Age (year)				<0.0001
<25	98	367	0.83 (0.63–1.10)	0.1969
25–35	181	564	1	
>35	162	260	1.94 (1.50–2.52)	<0.0001
Primary school at least				
No	342	842	1	
Yes	99	349	0.70 (0.54–0.90)	0.0059
Profit-making activity				0.6851
Farming/breeding/fishing/handicraft	187	489	1	
Employee status/shopkeeper	156	413	1.13 (0.85–1.50)	0.4058
No activity	98	289	1.11 (0.83–1.49)	0.4719
Number of children in the household				0.1303
1	75	265	1	
2	93	232	1.42 (0.99–2.01)	0.0527
3	95	229	1.47 (1.03–2.08)	0.0328
≥4	178	465	1.35 (0.99–1.84)	0.0554
Knows mosquitoes transmit malaria				
No	131	304	1	
Yes	310	887	0.75 (0.58–0.96)	0.0246
Knows mosquito net protection against malaria				
No	121	263	1	
Yes	320	928	0.81 (0.64–1.03)	0.0910

Factors associated with uncomplicated clinical malaria were grouped into information (in italic text) about (i) children aged 0–60 months, (ii) mosquito net, and (iii) mother or guardian of children characteristics. Univariate conditional logistic regression taking into account cluster effect was used

In the OKT health district (southern Benin), in univariate analysis, the variable SL was significantly associated with uncomplicated malaria episodes [OR 0.54 (95 % CI 0.42–0.70); p < 0.0001]. The use of an LLIN is lower in the case group than in the control. Other factors were significantly associated with uncomplicated malaria in the two districts. Age group (0–23 months) of children (p < 0.0001), mother’s or guardian’s level of education at least primary school (p = 0.0337), time the children went to bed the previous night (between nightfall and 10 p.m. and after 10 p.m.) versus at nightfall (p < 0.0001), and age of LLIN under 3 years (p = 0.0018) were uncomplicated malaria protection factors. Some factors were significantly associated with increasing of uncomplicated malaria episodes: (i) use of prophylactic treatments against malaria (p = 0.0063), (ii) use other anti-mosquito measures (p = 0.0003), (iii) age of mother, or guardian (≥35 versus 25–35, p = 0.0001), and (iv) number of children in the household (p = 0.0054; Table 1). In the DCO health district (northern Benin), in univariate analysis, the variable SL was significantly associated with decrease of uncomplicated malaria episodes [OR 0.63 (95 % CI 0.50–0.78); p < 0.0001] as it was for OKT district. Other factors significantly associated with decreasing of uncomplicated malaria were: (i) age group (0–23 months) of children (p < 0.0001), (ii) age of LLIN (under 3 years) (p = 0.0269), (iii) mother’s or guardian’s level of education at least primary school (p = 0.0059), and (iv) mother’s knowledge that “mosquitoes transmit malaria” (p = 0.0246). The use of prophylactic treatments against malaria (p = 0.0418) and mother’s or guardian’s age of mother, or guardian (≥35 vs 25–35, p < 0.0001) were associated with increasing of uncomplicated malaria episodes in DCO district (Table 2). In multivariate analysis, the variable SL was a significant protective factor against uncomplicated clinical malaria episodes, with PE = 0.41 (95 % CI 0.22–0.55) in the OKT district (Table 3). There was a significant interaction (p = 0.0233) between the effects of the mother’s or guardian’s level of education and the use of an LLIN in the DCO district. Among children whose mother had at least a primary school level of education, PE was 0.55 (95 % CI 0.28–0.72), and it was 0.16 (95 % CI −0.10 to 0.36) in the other children, suggesting a loss of effectiveness of LLIN when mothers or guardians of children had never attended school in the DCO (Table 4).

**Table 3:** Association between uncomplicated clinical malaria and sleeping under an LLIN adjusted for other variables, Ouidah–Kpomasse–Tori Bossito health district

Variables	Adjusted OR (95 % CI)
Sleeping every night under an LLIN for the 2 weeks before the survey	
Sometimes or never	1
Every night	0.59 (0.45–0.78)^a^
Children age group (month)	
24–60	1
0–23	0.31 (0.24–0.40)
Mother or guardian age group (year)	
<25	1.35 (1.03–1.78)
25–35	1
≥35	1.37 (1.07–1.75)
Mosquito net age group (year)	
>3	1
≤3	0.77 (0.61–0.97)
Use of drugs perceive to be prophylactic treatment against malaria	
No	1
Yes	1.29 (1.02–1.63)

Multivariate conditional logistic regression taking into account clustering effect was done

^a^Adjusted odds ratio (OR) on children age, mother age, mosquito net age, and using prophylactic treatment against malaria by children

**Table 4:** Association between uncomplicated clinical malaria and sleeping under an LLIN adjusted on other variables, Djougou–Copargo–Ouake health district

Variables	Adjusted OR (95 % CI)
Sleeping every night under an LLIN for the 2 weeks before the survey	
Sometimes or never	1
Every night	0.45 (0.28–0.72)^a^ 0.84 (0.64–1.10)^b^
Children age group (month)	
24–60	1
0–23	0.24 (0.19–0.31)
Mother or guardian age group (year)	
<25	1.02 (0.76–1.38)
25–35	1
≥35	1.41 (1.06–1.87)
Knowing mosquitoes transmit malaria	
No	1
Yes	0.71 (0.54–0.93)

Multivariate conditional logistic regression taking into account clustering effect was done

^a,b^Adjusted OR on children’s age, mother’s age and knowing mosquitoes transmit malaria

^a^Adjusted OR for group of children whose mother have been at primary school at least

^b^Adjusted OR for group of children whose mother have never been at school

## Discussion

This community-based case–control study was used to evaluate the effectiveness of LLIN in a context of wide distribution. When considering 1:3 case–controls, matching on the village of residence, “sleeping every night under an LLIN” conferred 40 % protection from malaria episodes in southern Benin (OKT) and up to 55 % in northern Benin (DCO) among children. The mother’s or guardian’s level of education was a significant modifier of the effectiveness of LLIN. This modification effect was only observed in the DCO district despite the similar frequency of mothers’ and guardians’ primary school level of education among controls in both districts: 32 % in the OKT and 29 % in the DCO. In the present study, use of an LLIN was low (17–57 %) and far from the 80 % expected and which can give mass effect [4, 22]. In addition, malaria vectors were resistant to pyrethroïd insecticides. The mortality rate of vectors after exposure to these insecticides was very low, under 90 % required [23]. Nevertheless, the PE of an LLIN was between 40 and 50 %. An LLIN can then contribute to reduce malaria case incidence as expected [4].

This study identified other factors significantly associated with uncomplicated clinical malaria. The main risk factors of malaria episodes were the use of drugs perceived to be malaria prophylactic treatments (herbal tea, paracetamol, and chloroquine) and the use of anti-mosquito measures other than bed nets (coil, domestic insecticide spray, and smoking with traditional grass). The use of herbal tea, paracetamol, and chloroquine for children as prophylactic drugs was very high. This habit can lead to temporarily diminution or disappearance of fever but not clean the parasites. In the same way, the use of anti-mosquito measures other than bed nets may reduce malaria vector biting but the risk of malaria infection and morbidity remains. However, the use of these non-recommended malaria prevention measures could be related to a perception of higher risk of malaria and therefore could be associated with the risk of clinical malaria.

Protective factors of uncomplicated malaria episodes were the child’s age >24 months, mother’s education level (at least primary school), and knowing of the role mosquitoes play in the transmission of malaria and of the protection of LLIN against malaria.

Social factor as school level has already known to be a determinant of malaria case occurrence [24, 25]. Other determinants that were not taken into account in this study, and related to the social and economic environment, probably interfered in the both districts. To better understand the determinants of the effectiveness of LLIN in an area, additional and specific anthropological studies are therefore needed.

Some limitations to this study stem from the use of the case–control study design. The efficacy of interventions deployed can be defective. For example, if malaria vectors are locally resistant to insecticides used for treated nets or for wall spraying, or *Plasmodium* are locally resistant to anti-malarial drugs used for preventive measures or chemoprophylaxis, the effectiveness of interventions may be affected in some areas more than others. The estimation of an average effectiveness, without taking into account several heterogeneous areas in terms of vector and parasite sensitivity, would not be relevant or would not detect localized deficiencies in effectiveness.

The different case definitions for uncomplicated malaria have advantages and disadvantages. The best definition of uncomplicated clinical malaria is based on a parasite density threshold associated with signs or symptoms suggesting malaria [18, 26]. The definition of clinical malaria cases in the present study was only based on the detection of *Plasmodium* infection by RDT, i.e. regardless of the parasite density. This definition could lack specificity [27]. Defining malaria attacks by the association of fever with a patent parasite infection (e.g., detected by a RDT) can overestimate the number of fevers caused by malaria (excess of false-positive results), even in areas of low endemicity. It may underestimate the PE of malaria interventions, especially as asymptomatic *Plasmodium* infections are more common in highly endemic areas than in areas of low endemicity and in the most immune compared to those who have not had exposure long enough to develop immunity. The use of parasite density to define malaria cases suitably at every level of endemicity and at all ages most often requires microscopy studies and therefore carries a higher cost and is a source of inter-study variability due to potential differences in reading blood smears. Although this definition avoids underestimating the effectiveness of LLIN and distortions in this estimation depending on malaria endemicity and the age of individuals, it does not reflect the burden of malaria as usually estimated by health systems.

This study has many valuable aspects nevertheless. The cases and controls were identified only in community concurrently from the same at-risk population. The health center recruitment may be a source of information bias, especially in terms of exposure variables. Since the LLIN were given at no cost, mothers may lie (prevarication bias) about their child’s exposure, especially in the health center area when the children were sick. In this study, direct observation of the net in the hung position in the house was used, knowing that questions about the use of mosquito bed nets could lead to a deliberate false response.

The study had a very short duration. The positive effect of short-term use cannot be found in longitudinal or cohort studies, which may change the community’s behavior. Given that individuals were asked the same questions several times, the quality of responses may be compromised, resulting in a high rate of LLIN use regardless of the child’s clinical status [18]. The consequence is no difference in exposure between individuals and therefore problems demonstrating the effectiveness of LLIN due to the loss of statistical power. Concerning the case–control study, the difficulties finding cases and controls according to epidemiological malaria endemicity and season may extend the duration of the study.

The risk of malaria attacks is assumed to be higher about 2 weeks after inoculation of sporozoites by anopheline vectors. The cover of LLIN can mean the possession and/or the hung of LLIN in the household [4, 18, 28]. The cover of LLIN can mean the possession and/or the hung of LLIN in the household. The first step before the use of LLIN (sleep under an LLIN) is logically its set up. In this study, it was assumed that the move of the net every morning and every night is little compatible with its correct use. Then, the definition of SL included the hung of net and the verification of its presence in the household. In most studies, the level of bed net use was estimated according to their use the night before in order to minimize memory bias [11, 29–33] as recommended by WHO indicators for the estimation of LLIN use [34]. The last definition can lead to information bias as an LLIN may be put away. An LLIN could also be use for needs other than those it was originally intended to.

A preliminary study conducted in 2008 in the OKT area showed an adjusted OR of 0.32 (95 % CI 0.15–0.71) within only six villages and a sample size limited to 35 cases and 115 controls [12]. In the context of the Gambian National Insecticide Bed Net Programme during the second year of the intervention, a case–control study was conducted to evaluate the program’s effectiveness. The adjusted OR of the association between net use and malaria morbidity was estimated at 0.41 (95 % CI 0.18–0.92), while the definition of the “use of a mosquito bed net” was not precise enough in terms of the frequency of use [9]. Another study was conducted in southern Colombia and considered a 1:3 case–control design with matching on village, age, and sex. The adjusted OR of the association between the impregnated net use the night before and malaria episodes was estimated at 0.44 (95 % CI 0.20–0.98), [11].

## Conclusion

In the context of a mass distribution of LLIN, their use still conferred protection in up to 55 % against the occurrence of clinical malaria cases in children. An LLIN stays then a relevant tool for malaria control. Social factors, the poor use and the poor condition of an LLIN can be in disfavour with its effectiveness. In areas, where LLIN coverage is assumed to be universal or targeted at high-risk populations, case–control studies should be regularly conducted to monitor the effectiveness of LLIN. The findings will help NMCP and their partners to improve the quality of malaria control taking into account the particularity of each area or region as far as possible.
